# Comparative genomics and virulome analysis reveal unique features associated with clinical strains of *Klebsiella pneumoniae* and *Klebsiella quasipneumoniae* from Trinidad, West Indies

**DOI:** 10.1371/journal.pone.0283583

**Published:** 2023-07-10

**Authors:** Aarti Pustam, Jayaraj Jayaraman, Adesh Ramsubhag

**Affiliations:** Department of Life Sciences, Faculty of Science and Technology, The University of the West Indies, St. Augustine, Trinidad and Tobago; Yenepoya University, INDIA

## Abstract

*Klebsiella pneumoniae* and *Klebsiella quasipneumoniae* are closely related human pathogens of global concern. The more recently described *K*. *quasipneumoniae* shares similar morphological characteristics with *K*. *pneumoniae* and is commonly misidentified as this species using traditional laboratory techniques. The vast mobilome in these pathogenic bacteria influences the dissemination of virulence factors in high-risk environments and it is, therefore, critical to monitor strains for developing effective clinical management strategies. Herein, this study utilized Illumina sequencing to characterize the whole genomes of nine clinical *K*. *pneumoniae* and one *K*. *quasipneumoniae* isolate obtained from patients of 3 major hospitals in Trinidad, West Indies. Reconstruction of the assembled genomes and implementation of several bioinformatic tools revealed unique features such as high pathogenicity islands associated with the isolates. The *K*. *pneumoniae* isolates were categorized as classical (n = 3), uropathogenic (n = 5), or hypervirulent (n = 1) strains. *In silico* multilocus sequence typing, and phylogenetic analysis showed that isolates were related to several international high-risk genotypes, including sequence types ST11, ST15, ST86, and ST307. Analysis of the virulome and mobilome of these pathogens showed unique and clinically important features including the presence of genes associated with Type 1 and Type 3 fimbriae, the aerobactin and yersiniabactin siderophore systems, the K2 and O1/2, and the O3 and O5 serotypes. These genes were either on or in close proximity to insertion sequence elements, phage sequences, and plasmids. Several secretion systems including the Type VI system and relevant effector proteins were prevalent in the local isolates. This is the first comprehensive study investigating the genomes of clinical *K*. *pneumoniae* and *K*. *quasipneumoniae* isolates from Trinidad, West Indies. The data presented illustrate the diversity of Trinidadian clinical *K*. *pneumoniae* isolates as well as significant virulence biomarkers and mobile elements associated with these isolates. Additionally, the genomes of the local isolates will add to global databases and thus can be used in future surveillance or genomic studies in this country and the wider Caribbean region.

## Introduction

*Klebsiella pneumoniae* (*K*. *pneumoniae*) is an opportunistic pathogen associated with nosocomial infections like pneumonia, meningitis, and urinary tract infections (UTIs) as well as community-onset infections like liver abscesses and endophthalmitis [1]. While antibiotic resistance critically affects the treatment of infections caused by *K*. *pneumoniae*, virulence is also a major player that contributes to the severity of infections [2].

Although classical *K*. *pneumoniae* (cKp) strains carried traits of virulence, reports on the acquisition of virulence genes influenced its importance on the effect and severity of infections [1, 3]. The discovery of hypervirulent *K*. *pneumoniae* (hvKp) in the 1980s drastically improved the significance of virulence in *K*. *pneumoniae* as agents responsible for liver abscesses and endophthalmitis [4]. Since then, hvKp has been reported worldwide and is considered dangerous, with the potential for metastatic spread in healthy individuals [5, 6]. Mucoviscosity is one feature that has been correlated with hvKp strains and can be differentiated from traditional cKp strains by the phenotypic-based string test (string ≥ 5mm) [7]. However, this is not a very reliable method since colony conditions and user techniques can easily influence the results [8]. While these features are rarely noted in cKp strains and appear to be unique to hvKp strains, they are associated with a significantly high mortality rate ranging from 3 to 42% in hvKp [5, 9]. It is also important to mention that uropathogenic *K*. *pneumoniae* (UPKp) also carries traits that play important roles in the persistence of UTIs [10].

Due to the significant heterogeneity in *K*. *pneumoniae* strains, virulence factors and secretion systems play different roles in pathogenicity [11]. Several virulence factors including those linked to adhesion, biofilm formation, capsular polysaccharide (CPS- K antigens), lipopolysaccharide (LPS- O antigens), and iron scavenging systems contribute to disease development and severity of infections by *K*. *pneumoniae*. Bacterial adhesion and biofilm formation are the two, first-step mechanisms of virulence, and they are mediated by the Type 1 and Type 3 fimbriae that are encoded by the *fim* and *mrkABCD* cluster of genes, respectively. A well-established characteristic of virulent *K*. *pneumoniae* is its ability to produce siderophores that scavenge iron from infected tissues in limited conditions [12]. Four siderophore systems are active in *K*. *pneumoniae*, namely enterobactin (*Ent*), salmochelin (*iro*), yersiniabactin (*ybt*), and aerobactin (*iuc*). The *Ent* system is typical of *K*. *pneumoniae* strains and is known to provide a limited supply of iron to the pathogen due to the hindrance of this system by lipocalin2 [11]. However, highly pathogenic strains are most often those that harbour the *iro*, *ybt*, and *iuc* systems that are commonly located in high pathogenicity islands [13].

The CPS and LPS are critical factors in the virulence of *K*. *pneumoniae* because they activate the host’s innate immune response and protect the bacterium against antimicrobial peptides, phagocytosis, and opsonization and can repress early inflammatory responses [11, 14, 15]. Currently, seventy-eight CPS and eight LPS are known. Of significant importance in hypervirulent variants of *K*. *pneumoniae* are K1, K2, K5, K16, K20, K54, K57, and KN1 [16], and the O1, O2, O3, and O5 serotypes that are linked to virulence and severe clinical infections [17]. Additionally, the hypermucoviscosity *rmpA* gene has been identified as a positive regulator of capsular synthesis in hvKp strains [18].

Secretion systems also play a vital role in bacterial pathogenesis and can be used at any point in the bacterial infection pathway. These can be used for the delivery of toxins to eliminate competitors, cell adhesion, and effector translocation into host cells [19, 20]. The Type I (T1), Type II (T2), and Type IV (T4) secretion systems are common in pathogenic bacteria like *K*. *pneumoniae* [21, 22]. However, the Type VI secretion system (T6SS) is a recent observation in *K*. *pneumoniae* species and is regarded as a versatile weapon due to its ability to secrete a wide range of effectors and toxins, thereby promoting infections [23].

Due to the challenges faced by pathological labs to distinguish the nature of virulent strains, whole genome sequencing (WGS) and comparative genomics have become powerful tools for genotyping and characterizing these strains [24]. The genome of the *Klebsiella* species mainly range from 5.2Mb to 5.6Mb in size, with an average GC percent of 57 [25]. Several comparative genomics approaches have been applied to characterize *Klebsiella* strains and provided important information on traits of clinical, epidemiological, and ecological significance. Genes for many of these traits of significant importance are commonly located on mobile genetic elements (MGEs) like plasmids, insertion sequences, and transposons, which are often vectors of horizontal transmission [26]. The close genetic relationship among members of the *Enterobacteriaceae* family such as *E*. *coli*, *Citrobacter spp*, and *K*. *pneumoniae* facilitates interspecies and intraspecies transmission via horizontal gene transfer, which has led to the emergence of strains with efficient characteristics that promote adaptation and general bacterial fitness [27].

Multi-locus sequence typing (MLST) is a popular genotyping method used to characterize relationships among bacterial strains and to track the global spread of resistant and virulent strains [28]. Through MLST, several sequence types (ST) have been linked to virulent *K*. *pneumoniae*, such as ST11, ST15, ST86, and ST307, which are either endemic or epidemic in some geographic regions including China and Europe [29, 30]. While there have been no reports of outbreaks linked to hvKp high-risk clones in the Caribbean, there has been one report of virulent strains belonging to ST11, ST15, and ST86 in this region [31].

Currently, there is a paucity of information on virulence factors and their associated MGEs in clinical *K*. *pneumoniae* isolates from Trinidad. This is the first comprehensive study to use a genomic approach to gain a deeper understanding of the genetic variations among clinical *K*. *pneumoniae* isolates originating from patients of three major hospitals in Trinidad, West Indies. We used comparative genomics to investigate the diversity and occurrence of virulence biomarkers in clinical hvKp, cKp, and UPKp isolates in order to bridge the knowledge gap of virulent *K*. *pneumoniae* in this country. This study’s findings would add important genome characteristics of clinical *K*. *pneumoniae* isolates from Trinidad to global databases and guide medical practitioners and policy makers in developing and implementing systems to aid in managing outbreaks of these pathogens.

## Materials and methods

### Ethics approval

Ethics approval was granted by the University of the West Indies, St. Augustine, Trinidad (CEC010/09/15), as well as the regional health authorities responsible for the management of the three hospitals included in the study. Participant consent was waived since samples were collected from the microbiology laboratories of the hospitals and there was no interaction with patients nor were their identities made available to any of the authors.

### Background and selection of the local clinical *Klebsiella* isolates, growth conditions, and genomic DNA extraction

The ten clinical isolates used in this study are from a larger 2015–2017 study [32] and represent varying combinations of resistance genotypes and phenotypes as shown in S1 Table (Origin of local isolates). The isolates were selected randomly from genotype-phenotype profiles using the INDEX and Random functions in Microsoft^®^ Excel^®^ (Version 2301). All the isolates were from clinical specimens including urine, sputum, and wound swabs, and originated from patients of 3 major hospitals in Trinidad, West Indies. Species identification, antibiotic resistance profiles, and virulence gene characterization of the isolates were previously reported [32]. While the *fimH* gene was present in most of the local isolates, there were differences in resistance phenotypes and genotypes of strains, e.g. some Non-ESBL/Carbapenemase producing isolates were found to contain ESBL genes using PCR [32] (See S1 Table). Of the ten selected isolates, nine were previously PCR confirmed as *K*. *pneumoniae* while one remained unidentified. The isolates were grown overnight at 30°C in Brain Heart Infusion Broth and the total genomic DNA was extracted using a modified CTAB protocol [33].

### Genome sequencing

Genomic DNA of the ten clinical isolates was sent to Novogene Corporation (USA) for whole genome sequencing using the Illumina HiSeq platform. The quality of the DNA was assessed using an Agilent 5400 Bioanalyzer, fragmented using sonification and the polished ends were ligated to Illumina adaptors that were amplified using index oligos P5 and P7. The amplified products were purified using the AMPure XP system, and the libraries were constructed using the NEBNext Ultra II DNA Library Prep Kit with an insert size of ~350 bp. Following this, the Agilent 2100 Bioanalyzer and qPCR were used to assess the size distribution and concentration of the libraries. Finally, the Illumina HiSeq platform (150bp PE to a depth of 1G) was used for whole genome sequencing, following which adapters and ligation sequences were removed, and the raw sequences were filtered to provide reads at a QC >30 and an error rate of ~0.03%.

### Whole genome assembly and annotation

Unless otherwise specified, the majority of the bioinformatic analysis was performed on the online server usegalaxy.eu [34]. The reads were checked for quality using FastQC (v0.73) and trimmed using Cutadapt (v4.0) to match a PHRED score ≥30. Shovill (v1.1.0) with the spades assembler enabled was used to assemble the surviving reads. To assess the quality of the assembled multi-fasta contig file, the Quality Assessment Tool for Genome Assemblies (Quast v4.6.0) [35] was used. Prokka (v1.14.6) and RAST (v2.0) [36] were used to annotate the multi-fasta contig file. The genomes were submitted to the NCBI database under Bioproject ID PRJNA752893.

### Species identification and genome completeness

Species identity was determined using The Microbial Genome Atlas (MiGA) [37], *Klebsiella pneumoniae* BIGSdb-Pasteur (https://bigsdb.pasteur.fr/klebsiella/), and Kleborate [38]. Genome completeness was predicted using MiGA and BUSCO (v5.3.2).

### *In silico* MLST and phylogenetic analysis

*In silico* Multilocus Sequence Typing (MLST) was performed using MLST v2.0 [39], which is hosted on the Center for Genomic Epidemiology (CGE) (http://www.genomicepidemiology.org/services/) website. CSI phylogeny [40] was used to identify Single Nucleotide Polymorphisms (SNPs) in the local genomes and reference *K*. *pneumoniae* (strains that had a similar ST to the local isolates), *K*. *quasipneumoniae*, and *K*. *variicola* genomes downloaded from the NCBI Reference Sequence Database (RefSeq). SNP variability was then represented in NgPhylogeny (https://ngphylogeny.fr/) using the FastTree/OneClick workflow with the built-in MAFFT alignment algorithm, BMGE alignment curation, and FastTree inference using a bootstrap method of 1000 replicates.

### Comparative genomics, characterization, and feature annotation of the local genomes

The local genome alignments were ordered against references NTUH-K2044 and KqPF26 using progressiveMauve [41]. Genomic features of the ten local genomes were obtained from Prokka and the RAST server. Roary (v3.13.0) was used to conduct pangenome analysis for core genes (>90%) and accessory genes (<50%) similarities. The core and accessory genes were functionally characterized using COG (Cluster of Orthologous Group) with the embedded EggNog Mapper (v2.0) [42], and KEGG (Kyoto Encyclopedia of Genes and Genomes) database.

Mobile elements were annotated using RAST, Prokka, and the IS Finder database [43]. Putative integrative and conjugative elements (ICE) were predicted using ICEfinder [44] and the result underwent blast analysis (blastn with an adjusted 90% similarity parameter) against the in-house database to determine closely related ICE. Plasmid-derived contigs and fragments of plasmids were identified using plasmidVerify [45] and PlasmidFinder (v2.1) [46]. The PHASTER tool [47] was used to determine the presence of intact/complete phages.

The ordered genome files from progressiveMauve were used to predict virulence factors against reference genomes NTUH-K2044, HS11286, and MGH 78578 using the Virulence Factor Database (VFDB) server (http://www.mgc.ac.cn/cgi-bin/VFs/genus.cgi?Genus=Klebsiella). Capsular serotype (CPS/ K antigen) and Lipopolysaccharides (LPS/ O antigens) were predicted using Kaptive [48]. Manually curated databases and blastn were used to determine specific virulence factors of interest, including the presence of the regulator of mucoid phenotype (*rmpA* and *rmpA2*) genes. Secretion systems and effector proteins were identified using TXSScan MacSYFinder (v1.0.5), SecRet4 [49], and SecRet6 [50]. Circular plots of the gene coordinates of the virulence factors and secretion systems of the *K*. *pneumoniae* genomes were generated in the CgView server [51]. All heatmaps were generated using TBtools (v1.09) [52] and synteny maps were generated using Gene Graphics [53].

## Results

### Genome identity and general statistics

The reconstructed sequences of the local genomes were confirmed as *K*. *pneumoniae* (H1_6, H1_20, H1_36, H2_26, H2_41, H2_55, H2_81, H3_42, and H3_66) and *K*. *quasipneumoniae* (H1_16) via rMLST, MiGA, and Kleborate, and had >99% ANI (MiGA) to published strains. The genome sizes ranged from 5.4 to 5.6Mb. The local genomes were represented by 39 to 70 contigs (≥500bp) with a maximum contig length from 505,964 to 1,082,608 bp and a minimum contig length from 201 to 223 bp. The GC% ranged from 56.74 to 57.83%, which was similar to the references NTUH-K2044 (57.39%) and KqPF26 (57.84%). The genomes were estimated to be 98.4% complete (BUSCO), with less than 1% contamination (removed before downstream analysis), and had an estimated coverage of 209x to 266x. A total of 5,111 to 5,835 coding genes were predicted, with several rRNA genes (5s, partial16s, 23s) on multiple contigs. The overall statistical features of the ten sequenced genomes are shown in Table 1.

**Table 1:** Draft de-novo assembled genome statistics of nine *K*. *pneumoniae* species and one *K*. *quasipneumoniae* species isolated from patients who utilized major hospitals in Trinidad, West Indies.

Species: *K*. *pneumoniae*										Species: *K*. *quasipneumoniae*
Strain	H1_6	H1_20	H1_36	H2_26	H2_41	H2_55	H2_81	H3_42	H3_66	H1_16
**NCBI Genome Accession**	JAIEZC000000000	JAIEZE000000000	JAIEZF000000000	JAIEZG000000000	JAIEZH000000000	JAIEZI000000000	JAIEZJ000000000	JAIEZK000000000	JAIEZL000000000	JAIEZD000000000
**Size (Mb)**	5.4	5.5	5.6	5.4	5.5	5.4	5.4	5.4	5.6	5.4
**GC (%)**	57.14	57.12	56.93	57.26	57.19	57.32	57.32	57.23	56.74	57.83
**Estimated coverage (X)**	226	235	223	265	266	227	253	239	209	227
**Contig number (≥500 bp)**	59	62	60	53	70	39	57	46	62	51
**Max/min contig length (bp)**	539,484/204	661,598/205	739,852/204	512,276/202	505,964/201	719,236/202	559,001/202	1,082,608/223	739,856/205	920,740/205
**Contig N50 (bp)**	346,604	302,493	304,658	307,999	270,013	436,854	265,046	474,974	282,908	460,957
**Total genes**	5,382	5,553	5,596	5,403	5,609	5,364	5,420	5,404	5,630	5,333
**Total CDS**	5,259	5,421	5,467	5,277	5,493	5,244	5,300	5,294	5,502	5,207
**Coding genes**	5,143	5,321	5,350	5,190	5,403	5,146	5,201	5,198	5,385	5,111
**RNA genes**	123	132	129	126	116	120	120	110	128	126
**rRNA**	6, 9, 12 (5s (4 partial), 16s (partial), 23s (partial))	9, 10, 15 (5s (7partial), 16s (partial), 23s (partial))	8, 11, 16 (5s (6 partial), 16s (partial), 23s (partial))	6, 12, 11 (5s (4 partial), 16s (partial), 23s (partial))	7, 7, 11 (5s (5 partial), 16s, 23s (partial))	8, 8, 11 (5s (5partial), 16s (partial), 23s (partial))	8, 7, 9 (5s (6partial), 16s (partial), 23s (partial))	5, 8, 4 (5s (3 partial), 16s (partial), 23s (partial))	8, 9, 17 (5s (6 partial), 16s (partial), 23s (partial))	9, 9, 15 (5s (7 partial), 16s (partial), 23s (partial))
**tRNA**	84	89	84	84	80	84	80	82	83	85
**ncRNA**	12	9	10	13	11	9	16	11	11	8
**Total Pseudogenes**	116	100	117	87	90	98	99	96	117	96
**MLST/CC**	ST 111/ CC 0	ST 194/ CC 137	ST 15/ CC 0	ST 307/ CC 0	ST 11/ CC 0	ST 86/ CC 0	ST 280/ CC 0	ST 528/ CC 0	ST 15/ CC 0	ST 283/ CC 31

**Fig 1:**
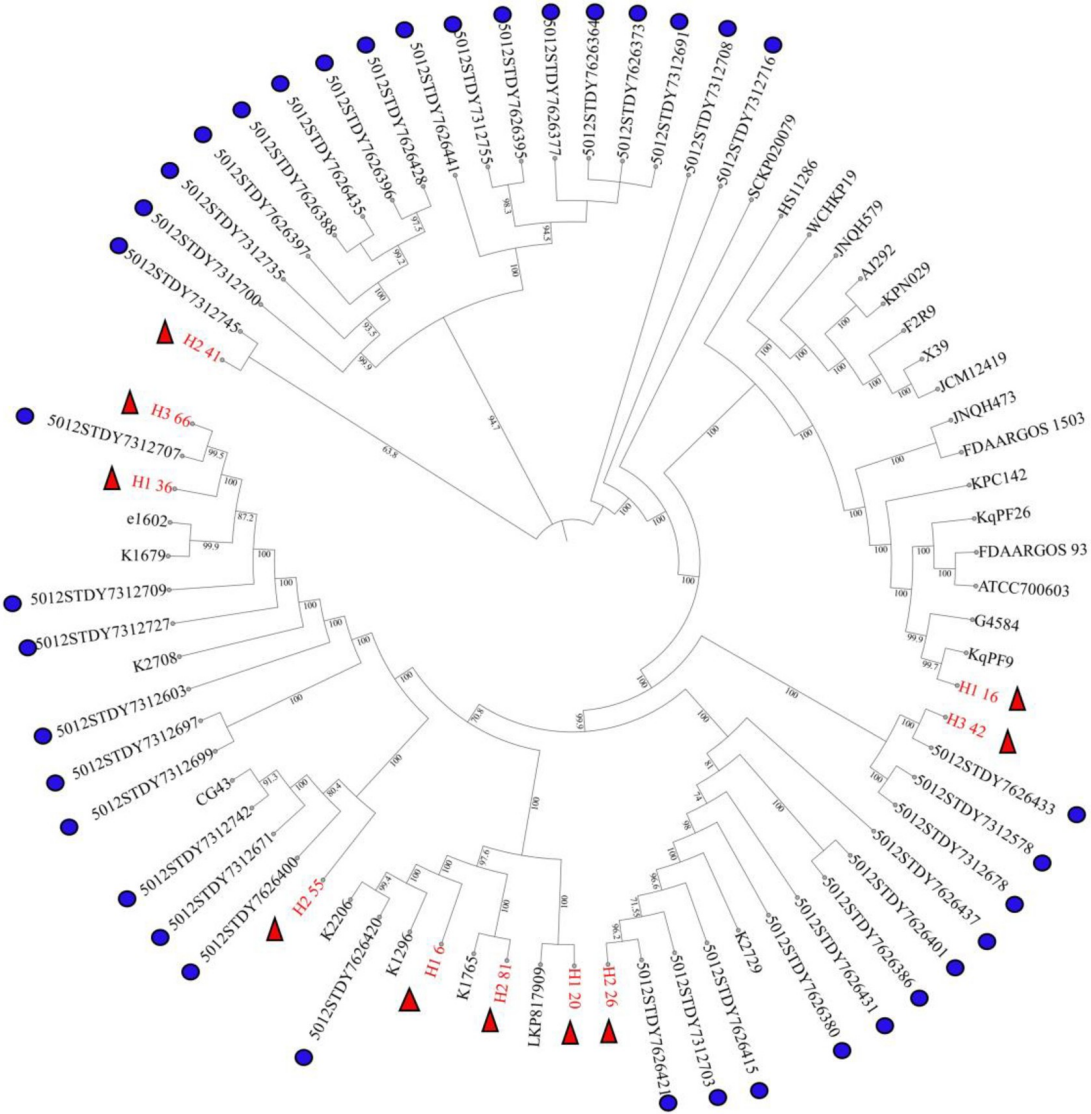
Phylogenetic reconstruction of wgSNPS from nine *K*. *pneumoniae* and one *K*. *quasipneumoniae* clinical isolates. The phylogenetic tree was built using the FastTree/OneClick workflow with the built-in MAFFT alignment algorithm, BMGE alignment curation, and FastTree inference using a bootstrap method of 1000 replicates. The local isolates are highlighted with the red triangle symbol. The Caribbean reference strains are highlighted with the blue circle symbol.

To establish the genomic population structure, the local isolates were assigned to STs based on the presence and allelic variations in seven housekeeping genes in the genomes (Table 1). Nine different STs were predicted including ST11, ST15, ST86, ST111, ST194, ST280, ST283, ST307, and ST528. To explore the diversity of the local isolates, wgSNP analysis was used to investigate the phylogeny of the ten *Klebsiella* genomes and forty-nine references (See S1 Table for the origin of the reference strains). As seen in Fig 1, there was a mix of clinical isolates from Trinidad that are similar to global and Caribbean reference strains based on SNP variation predicted by CSI phylogeny. For instance, local isolate H2_81 was highly similar to reference strain K1765, both of which had the same ST and the least SNP variation when compared to the other isolates and references (See S1 Table for SNP matrix). In comparison, isolates such as H2_55and H1_6 were found in similar clades to reference with the same ST but did not directly cluster with the references possibly due to a higher SNP variation between the local isolates and references. One of the local isolates, H3_66, of ST15 appeared to be closely related to the Caribbean reference 5012STDY7312707 and only varied by 28 SNPs compared to H1_36, also of ST15, but had more SNP variation of 44 with the Caribbean reference and 52 with H3_66.

### Genomic synteny analysis

**Fig 2:**
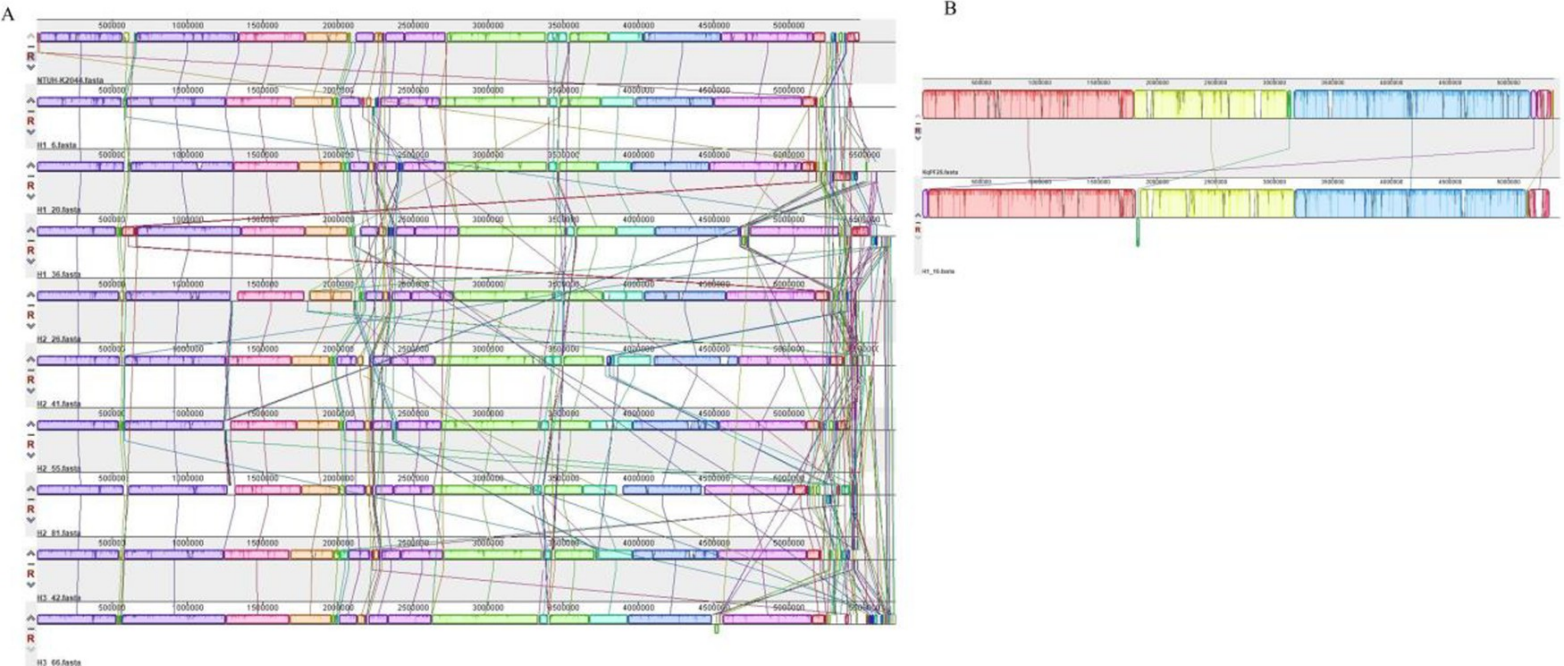
Progressive mauve alignment of nine local *K*. *pneumoniae* genomes against the reference NTUH-K2044 (A) and a local *K*. *quasipnuemoniae* genome against the reference KqPF26 (B). Lines connecting identical colored modules represent the LCB among the genomes without rearrangements while the white areas within a block represent non-homologous regions. Incomplete/ white blocks indicate that there was no alignment between the genomes.

Progressive Mauve alignment was used to generate the ordered synteny (Mauve) between the local genomes and reference genomes of *K*. *pneumoniae* (NTUH-K2044) and *K*. *quasipneumoniae* (KqPF26), respectively. Generally, the genomic synteny of the *Klebsiella* species displayed many local collinear blocks (LCBs). It appeared that the local *K*. *pneumoniae* genome has smaller LCBs or similar regions (Fig 2A) compared to *K*. *quasipneumoniae* (Fig 2B). The color and position of lines indicated that there are some regions of rearrangement in the local isolates. While the genome is in its draft form and smaller LCBs relating to smaller contigs are not usually significant, the haphazard localization of these in some genomes also indicated levels of genome rearrangement not shared with the reference.

### Pangenome analysis, COG, and KEGG functional annotation

The pangenome of the local *K*. *pneumoniae* isolates consisted of 8534 gene clusters compared to the local *K*. *quasipneumoniae* isolate that only had 5557 gene clusters. The pangenome was divided into core genes (>90% of the local genomes) and accessory genes (<50% of the local genomes). The core genes were attributed to 50.7% of the *K*. *pneumoniae* gene clusters and 80.6% of the *K*. *quasipneumoniae* gene clusters, while the accessory genes comprised up to 43% of the *K*. *pneumoniae* gene clusters and 10.4% of the *K*. *quasipneumoniae* gene clusters (Table 2). Overall, less than 10% of the genomes were unclassified.

**Table 2:** Pangenome analysis of clinical *K*. *pneumoniae* and *K*. *quasipneumoniae* isolates.

Species	*K*. *pneumoniae*	*K*. *quasipneumoniae*
**Pangenome**	8534	5557
**Core genes**	4323	4478
**Accessory genes**	3668	578
**Unclassified**	543	501

The *Klebsiella* species core and accessory protein-coding genes were functionally categorized based on COG and KEGG criteria. Nineteen of the twenty-six COG functional categories (S1A Fig) and six KEGG Orthology categories (S1B Fig) were assigned to the gene clusters. COG functionally annotated most of the core and accessory gene clusters, with less than 1.5% of the core genes remaining unassigned. Eight assigned COG categories of the core genes of both *K*. *pneumoniae* and *K*. *quasipneumoniae* not only had a greater abundance of gene clusters than the accessory genes but overall, also accounted for a larger proportion of the gene clusters. Additionally, approximately 2300 and 2600 of the core gene clusters, and 463 and 837 of the accessory gene clusters of the *K*. *pneumoniae* and *K*. *quasipneumoniae* isolates, respectively, were assigned to KEGG pathways. It appeared that while COG inadequately linked defense mechanisms in the local *Klebsiella* species, KEGG assigned the local isolates to human disease pathways and was predicted to be more involved in infectious diseases (bacterial pathways).

### Mobilome analysis

**Fig 3:**
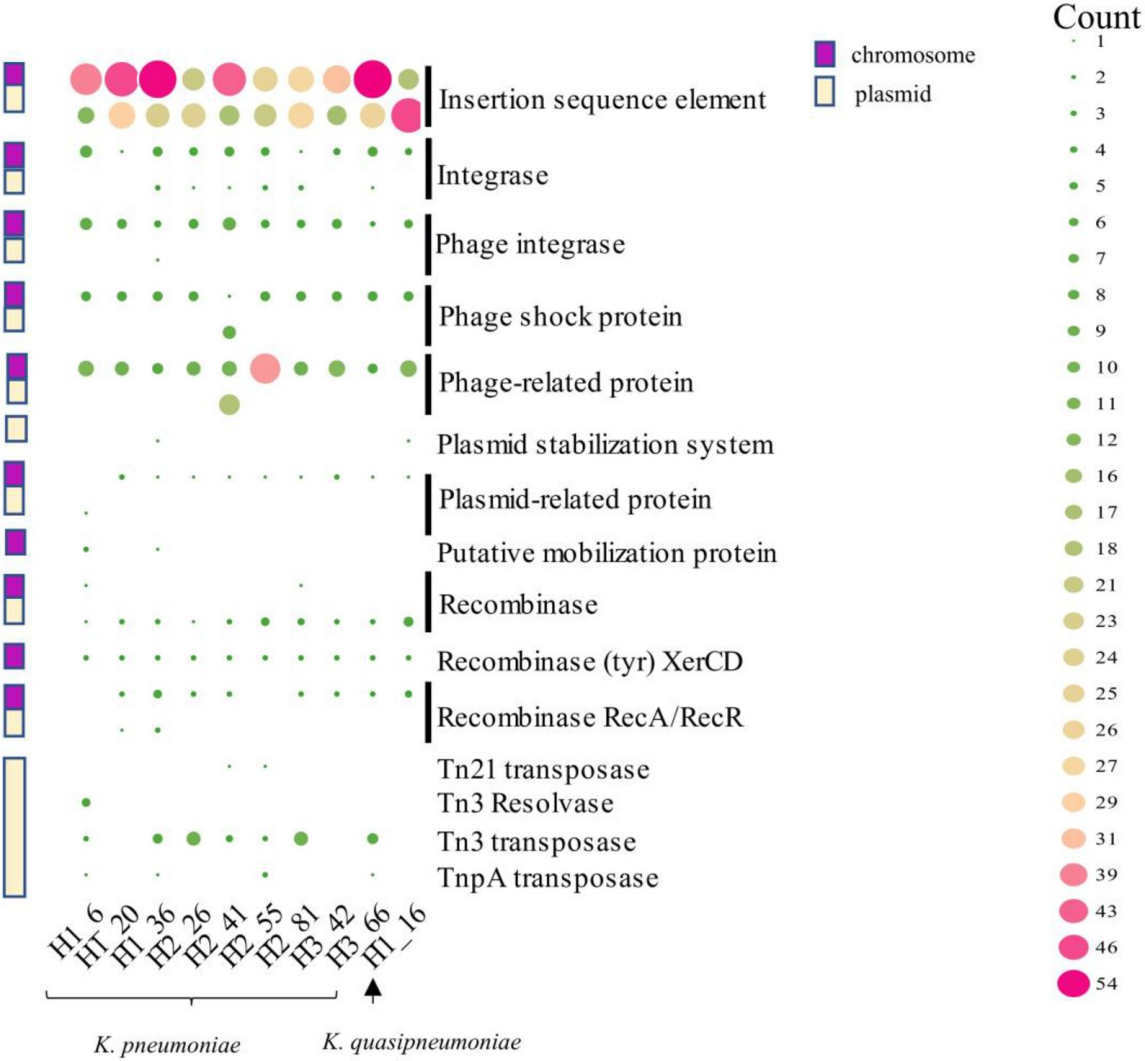
Distribution of mobile elements on chromosomal and plasmid-derived contigs among local clinical *K*. *pneumoniae* and *K*. *quasipneumoniae* isolates.

MGEs were annotated using a combination of Prokka, RAST, and mobile element databases. MGEs in the form of plasmids and insertion sequences, or genes associated with phage-related proteins, integrases, transposases, Tn proteins, and resolvase, were distributed among the local isolates as seen in Fig 3. In general, a total of 880 MGEs were detected among the local isolates. The MGEs of the *K*. *pneumoniae* isolates were mostly located on chromosomes (n = 535) rather than plasmids (n = 272). The local *K*. *quasipneumoniae* isolate maintained an almost equal distribution of MGEs on chromosomes (n = 43) and plasmids (n = 30). IS elements represented the majority of MGEs which were distributed among 16 IS families in the local *Klebsiella* isolates (S2 Fig). Of the IS families predicted, the IS3 family had the most IS elements (n = 134) which further consisted of 24 IS groups. Additionally, truncated fragments from thirteen different plasmids were identified with >95% similarity to reference *K*. *pneumoniae* and *K*. *quasipneumoniae* genomes (See S2 Table for plasmids predicted). Of these plasmids, the IncFIB(K) was the most common in the genomes, except H2_55. Other commonly noted plasmids were IncFII(K) and IncR. Several intact phages including *Salmon Fels*, *Klebsi phiKO2*, *Klebsi ST15 OXA48phi14*.*1*, *Entero mEp237*, and *Escher 500465* were also predicted and had a 100% identity to reference *Enterobacteriaceae* strains (See S2 Table for phages predicted).

### Virulome analysis and secretion systems

**Fig 4:**
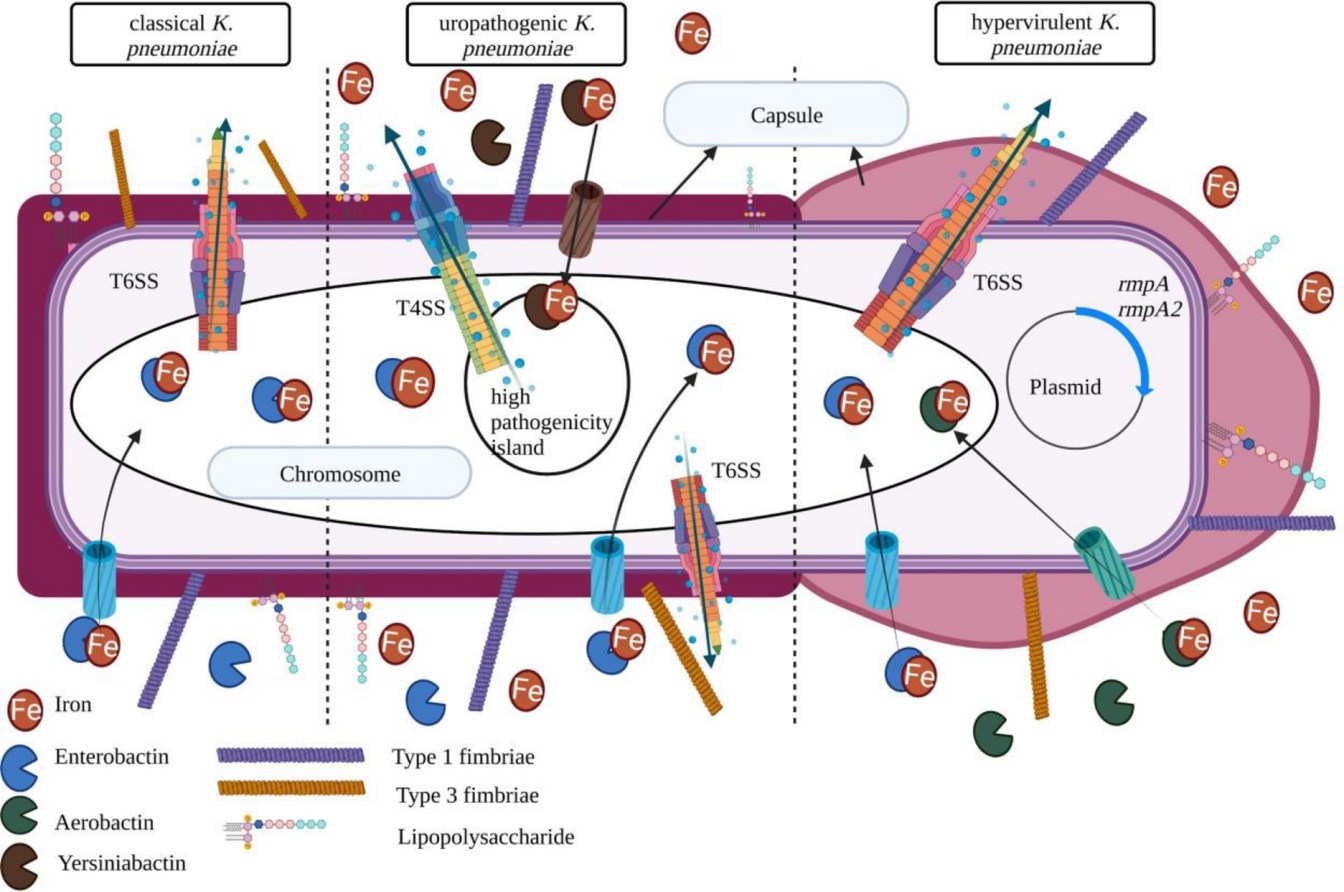
Virulence factors associated with local cKp, UPKp, and hvKp. The diagram was generated in BioRender.com.

The local strains displayed a myriad of virulence factors, some of which were unique to the UPKp (H1_6, H1_20, H2_41, H2_81, and H3_42), and hvKp (H2_55) isolates when compared to the cKp (H1_36, H2_26, and H3_66) isolates and the classical *K*. *quasipneumoniae* (cKqp) (H1_16) isolate (Fig 4). In addition to unique virulence features, the UPKp isolates harbored the uropathogenic specific protein, *usp*.

**Fig 5:**
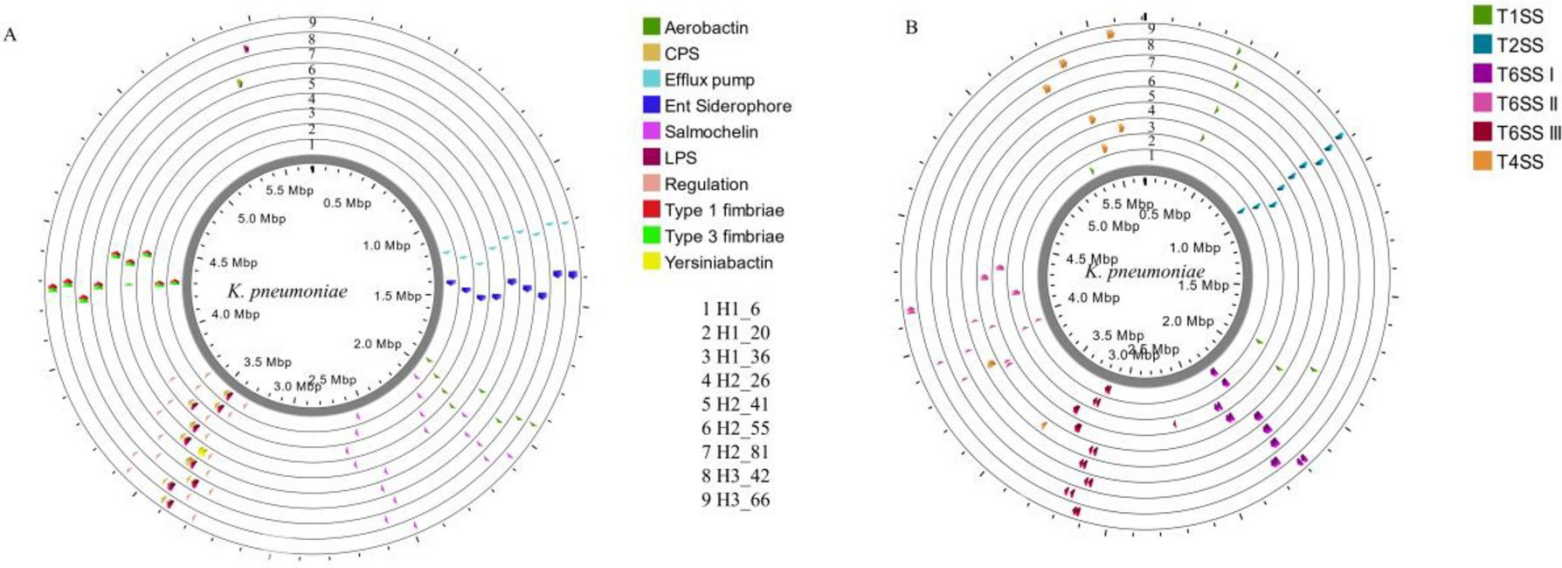
Mapped coordinates of genes associated with virulence factors (A) and secretion systems (B) in local *K*. *pneumoniae* isolates.

The genomes of the *K*. *pneumoniae* isolates were ordered against the complete reference genome NTUH-K2044 and the genome location of genes associated with virulence factors and secretions systems are highlighted in Fig 5A and 5B, respectively. The genes linked to virulence factors included those that contributed to adherence, antiphagocytosis, iron uptake, serum resistance, and regulation, and were within a similar region in the genomes, with the exception of the *iuc* cluster of genes and the CPS (K2 serotype) region in the local isolate H2_55 (ring 6 in Fig 5A).

The Type 1 fimbriae (*fimA–fimK*) and the Type 3 fimbriae (*mrkABCD*) cluster of genes responsible for the adherence and biofilm formation of the *Klebsiella* species were detected in the *K*. *pneumoniae* and the *K*. *quasipneumoniae* isolates (See S3 Fig for general organization of genes). It is of note that IS elements and the *marR* transcriptional regulator were found upstream of the Type 1 fimbriae in the local isolates.

While it is common to observe the *Ent* siderophore system in *K*. *pneumoniae* genomes, genes from the other three siderophore systems were also detected in the local isolates (See S3 Table for genes associated with siderophores). An incomplete *iro* system was detected, with only the *iroE* and *iroN* being observed in the local isolates. Furthermore, the hvKp H2_55 isolate was the only local *K*. *pneumoniae* with a complete *iuc* pathogenicity island of genes. Another outstanding observation was the prevalence of a complete *ybt* system in the local isolate H2_41. Apart from adherence, biofilm formation, and iron exchange, other genes, and systems that are also associated with virulence including the *focA* (formate transport), *zapA* (cell division), *satP* (succinate acetate/proton symport), and the iron transport operon *feoABC* were also present in all the local isolates.

Additionally, genes that are responsible for the capsule synthesis (CPS locus) and serum resistance (*rfb* locus) prevailed in the local isolates and were predicted to putatively form genomic islands. It should be noted that all the isolates displayed varying CPS loci (See S3 Table for serotypes). Of interest, is H2_55 which not only had the K2 CPS serotype, but also the O1/O2 Variant 1 *rfb* cluster (S3 Fig). Other *rfb* clusters including O1/O2 Variant 2, O3b, and O5, were present in the local genomes, and the differences in the genes that manage these clusters are indicated in S3 Fig. It is noteworthy to mention that H2_55 was the only isolate that had the regulation mucoid, *rmpA* and *rmpA2*, genes which were flanked by the MGE IS1N.

**Fig 6:**
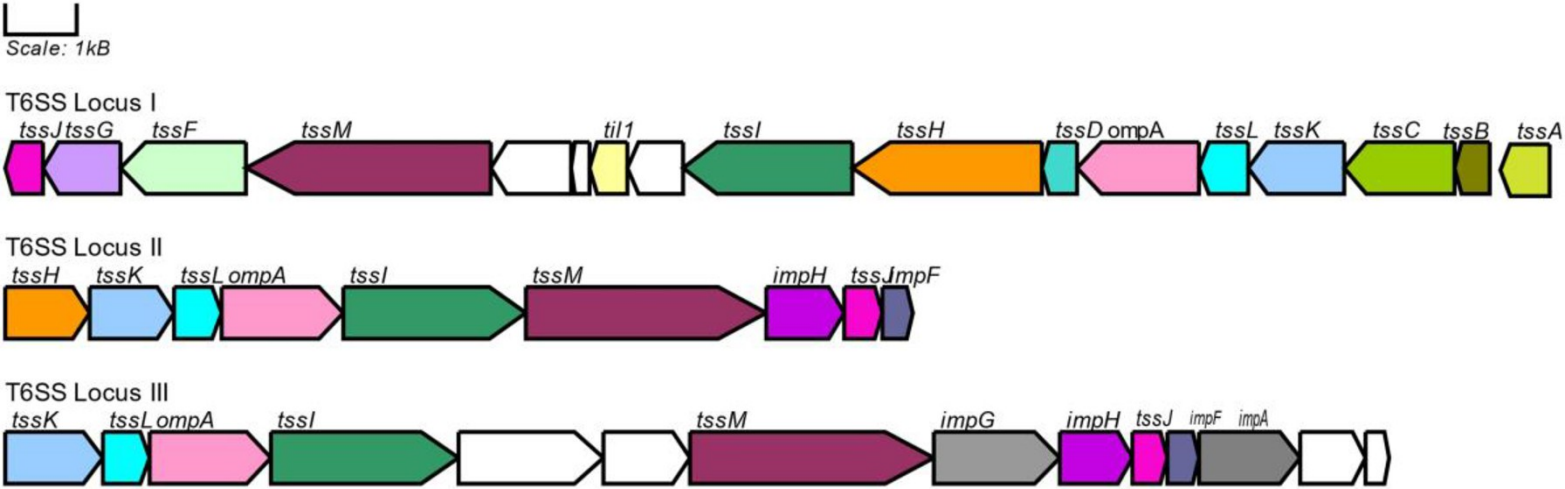
Organization of the T6SS in local isolate H1_36.

Secretion systems are another factor that promotes virulence and antibiotic resistance. The local isolates had genes for multiple secretion systems including T1SS, T2SS, T4SS, and the more recently described T6SS, which were observed to be located in similar regions of the genomes (Fig 5B). The two-partner passenger translocator (T5bSS) of the T5SS was noted in all the local isolates. Local isolates H1_20, H2_26, and H2_55 had the complete cluster of genes responsible for the organization of the T1SS (S3 Table secretion system, rings 2, 4, and 6 in Fig 5B). The other local isolates had the *omf* gene further apart from the *abc* and *mfp* genes and can account for the differences in the location of the T1SS in the local isolates. The T4SS comprising the general secretion pathway proteins was noted in all the local isolates in relation to an IncF plasmid. In addition, local isolate H2_41 also had the T4SS *virB* cluster of genes which was in close proximity to the IncQ plasmid. Genes from the 3 conserved loci of the T6SS were found in the *K*. *pneumoniae* isolates and their general organization in the local isolates is highlighted in Fig 6. The *tssB-M* genes that generate the core component of the T6SS were present in the isolates. The *vrgG*, *Hcp*, and *PAAR* effector components and putative effector (Table 3) and immunity (Table 4) proteins were also identified within the conserved loci as well as in other regions within the genome. It is also worth mentioning that while the PhoPQ pumps were present in all the genomes, the conserved T6SS regions of isolates H1_6 and H1_36 were flanked by these pumps.

**Table 3:** T6SS effector proteins detected in local clinical *K*. *pneumoniae* isolates.

Isolate	Isolate orf	% Identity	Effector Protein	Effector Type	Referenced Literature (effector proteins and function)
**H2_41**	orf1805	92	EFF01453	Tse	54
**H2_55**	orf1870	96			
**H2_81**	orf5027	92			
**H1_36**	orf1887	80			
**H2_26**	orf3500	90	EFF01467	Tde	55
**H2_26**	orf3049	81	EFF01826	Tse	56
**H1_6**	orf2962	80			
**H1_36**	orf3071	80			
**H2_41**	orf2953	80			
**H2_55**	orf2950	80			
**H3_66**	orf2875	80			
**H1_20**	orf2057	100	EFF18621	Tle	57
**H2_26**	orf2185	96	EFF00560	-	58
**H2_26**	Orf3521	94			
**H1_6**	orf2114	96			
**H1_20**	orf2050	96			
**H1_36**	orf2175	96			
**H2_41**	orf2060	96			
**H3_42**	orf2092	96			
**H3_66**	orf1979	96			

The data in this table was generated using SecRet6 (http://db-mml.sjtu.edu.cn/SecReT6/) T6SS prediction tool, with an 80% blast identity threshold value. The ‘-‘ indicates that the effector type is uncharacterized.

**Table 4:** T6SS immunity proteins detected in local clinical *K*. *pneumoniae* isolates.

Isolate	Isolate orf	% Identity	Immunity Protein	Cognate Effector	Referenced Literature (immunity proteins and functions)
**H1_6**	orf4535	93	IMU00344	EFF01520	59
**H1_20**	orf4481	93			
**H1_36**	orf4729	93			
**H2_55**	orf4554	93			
**H2_81**	orf4464	93			
**H3_42**	orf4526	93			
**H3_66**	orf4535	93			
**H2_26**	orf2191	97	IMU17297	EFF18621	57
**H1_20**	orf2055	95			
**H2_41**	orf2064	100			
**H2_41**	orf2067	96			
**H2_55**	orf2053	97			
**H2_81**	orf2069	95			
**H2_26**	orf2195	98	IMU17298		
**H1_20**	orf2056	91			
**H2_41**	orf2065	100			
**H2_55**	orf2054	90			
**H2_26**	orf2194	99	IMU17299		
**H1_20**	orf2054	96			
**H2_41**	orf2066	100			
**H2_81**	orf2068	96			
**H3_42**	orf2098	100			
**H3_42**	orf2099	92			
**H2_26**	orf2190	94	IMU17300		
**H2_26**	orf2196	94			
**H1_20**	orf5223	99			
**H1_20**	orf5226	92			
**H2_41**	orf2068	100			
**H2_55**	orf2052	94			
**H2_81**	orf2070	94			
**H3_42**	orf2097	99			

The data in this table was generated using SecRet6 (http://db-mml.sjtu.edu.cn/SecReT6/) T6SS prediction tool, with an 80% blast identity threshold value. The ‘-’ indicates that the effector type is uncharacterized.

**Fig 7:**
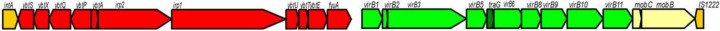
Genomic island displaying the high *ybt* pathogenicity island and T4SS in the local clinical *K*. *pneumoniae* isolate H2_41.

Interestingly, isolate H2_41 contained a genomic island (~52kb) that consisted of the T4SS *virB* cluster of genes and the high *ybt* pathogenicity island (~32kb) (Fig 7). The mobile element prophage integrase, *intA*, flanked the *ybt* pathogenicity island, and downstream of the T4SS were mobilization proteins, *MobB* and *MobC*, as well as transposase IS1222 from the IS3 family. It is worth noting that this genomic island was predicted to be present on a putative ICE, which when blasted against the ICEberg database was similar to *E*. *coli EDA1* and *K*. *pneumoniae HS11286*.

## Discussion

Genomic plasticity is a major factor in the spread of virulence and antibiotic resistance in *K*. *pneumoniae* [26]. While antibiotic resistance in *K*. *pneumoniae* is significantly important in clinical settings and the resistome of the local isolates is the focus of another ongoing study, virulence serves as an important factor that is used to enhance the invasiveness and persistence of infections [2]. We used WGS, a top Next-generation sequencing approach, to investigate the genomic content of nine clinical *K*. *pneumoniae* isolates and one clinical *K*. *quasipneumoniae* isolate obtained from patients at three major hospitals in Trinidad, West Indies. Several bioinformatic tools were used to investigate the virulome, mobilome, secretome, and phylogeny of each genome. We also used the assembled reads of the local genomes to validate the species identity of the isolates via the presence of the seven housekeeping genes. The nine *K*. *pneumoniae* isolates were confirmed as previously predicted [32], while the isolate that was inaccurately characterized as *K*. *pneumoniae* using clinical laboratory methods was identified as *K*. *quasipneumoniae*. This finding is similar to another study where clinical isolates identified as *K*. *pneumoniae* in diagnostic microbiology laboratories were later determined to belong to *K*. *quasipneumoniae* [54]. This was not surprising since accurate identification of members of the *Klebsiella* species can be challenging for most hospital laboratories since members of this genus, especially *K*. *pneumoniae*, *K*. *quasipneumoniae*, and even *K*. *variicola* share similar colony morphology and biochemical properties [55]. However, the findings of this study demonstrate the need to implement molecular techniques targeting specific housekeeping genes to properly identify *Klebsiella* species at the local hospital laboratories. Although the main focus of this study was on clinical *K*. *pneumoniae*, the *K*. *quasipneumoniae* isolate was also included in downstream analysis due to the growing concern for this species in the clinical world [56].

The size and the GC% of the local *K*. *pneumoniae* and the *K*. *quasipneumoniae* genomes were similar to reference strains. Several local isolates were classified into ST groups that contain international high-risk clones including ST11, ST15, and ST307 associated with major epidemics [57]. In order to get a better insight into the diversity of the local isolates, we performed wgSNP analysis with references to similar STs including isolates from a recent Caribbean study [31], the United Kingdom, Taiwan, and Ireland [58]. The data revealed that there is a wide assortment of isolates present in Trinidad, some of which clustered with global and Caribbean references. Despite the fact that this country took part in the Pilot Caribbean study [31], it is not possible to say whether the isolates from this current study are closely related to reference Caribbean isolates originating in Trinidad. Nonetheless, we suggest the possibility of the presence of transmission networks and therefore, emphasize the importance of identifying putative reservoirs of *K*. *pneumoniae* that may be involved in the transmission of this pathogen between hospitals within this country and eventually the ability to encourage global dissemination. While some of the local isolates, for instance, cKp H1_36, UPKp H1_6, and hvKp H2_55, clustered in clades with references, it is crucial to note that these isolates were not identical to any reference isolates, which highlights the diversity of *K*. *pneumoniae* isolates in this country.

Although the majority of the local isolates were attributed to unique STs, wgSNP groupings appear to be linked to acquired metabolic functions based on COG and KEGG analysis as many isolates shared a large core genome and thus indicated the genomic plasticity that is contained within this genus. MGEs are essential in the transmission of virulence genes within and between species. The virulence genes and secretion systems were inserted between or on MGEs. IS elements dominated in the local isolates and although they are the simplest mobile elements found in bacterial pathogens they are critical in the dissemination of virulence and resistance genes via horizontal gene transfer. It was not unexpected that we observed members of the IS3 family flanking the virulence genes in the local genomes since these IS elements are commonly reported in virulent *K*. *pneumoniae* [59, 60]. Additionally, similar to other published reports, this study also noted the prevalence of *Klebsi phiKO2 and klebsi ST15 OXA48phi14*.*1* phages in the local genomes [24, 61] which was not unexpected since they constitute a major player in the virulence and evolution of important pathogenic bacteria [62].

Virulence factors play an essential role in determining the severity of infection caused by *K*. *pneumoniae* and, hence, are often used to characterize strains of this pathogen. Based on our initial study [32] virulence genes were prevalent in the local isolates according to PCR. However, WGS of selected isolates allowed us to perform a more in-depth analysis utilizing bioinformatics tools to investigate the virulome of the isolates to putatively determine the pathogenicity of the local isolates and hence their potential effect in clinical settings of this country. While the ten isolates had potential virulence factors, local hvKp and UPKp isolates stood out for carrying unique features that may have the ability to encourage severe infections.

Similar to another study, the local isolates’ Type 1 and Type 3 fimbriae were homologous to the conserved gene cluster *fim-pecS-pecM-mrk* [63]. The Type 1 fimbriae are critical in initiating the adhesion process of the bacteria to the host, and while it is necessary if cKp and cKqp isolates were to contribute to virulence, it has been hypothesized to enhance virulence in UPKp [64]. The Type 3 fimbriae are critical for biofilm formation of uropathogenic strains, as well as nosocomial strains, and are functionally expressed once the 6 *mrk* genes are observed [65] as was seen in the local isolates. Although the *pecS* and *pecM* proteins are members of the *MarR* transcriptional regulators of virulence genes, and it is common that the Type 1 and Type 3 fimbriae are found within the conserved pathway that includes the *MarR* proteins, it has been shown that these proteins are often dispensable in lung infections caused by *K*. *pneumoniae* [66]. It is also noteworthy that the fimbriae conserved pathway was flanked by the ISEcp1 transposase in the local isolates, which therefore suggests the potential mobility of these virulence factors. Apart from the fimbriae, the local isolates also carried iron scavenging systems which are imperative for the uptake of iron during limiting conditions. Furthermore, the presence of the *feoABC* transporters in the local isolates suggests that iron homeostasis is maintained in these pathogens [67] and therefore increases their chances of survival in limiting conditions.

We also observed biomarkers of significant importance that can putatively influence infections due to *K*. *pneumoniae* in the local hvKp isolate. In particular, the *iucABCD* cluster of genes that forms the *iuc* siderophore system was unique to the hvKp isolate. Although the hvKp can produce the four siderophores, the *iuc* system accounts for more than 90% of siderophore production and is critical for growth/survival ex vivo and for extreme virulence in vivo [68]. Additionally, this isolate also had the K2 and the O1/O2 Variant 2 serotypes. Apart from these serotypes dominating invasive human infections, they have also been observed in ST86 virulent isolates from a Pilot study in the Caribbean [24, 69]. Also, it was not unexpected that the local hvKp isolate had the *rmpA* gene that is responsible for K2 synthesis since this gene is particularly linked to hvKp strains and is often involved in invasive purulent diseases and liver abscesses [24, 70]. While the hypervirulent isolate from this study was obtained from a patient who was warded at the National Organ Transplant Unit in the country, and it was not surprising that the traits of hypervirulence were observed, the corroborating data on the specimen at the time of collection did not specify links to any specific disease. However, based on the basic background information on the host of this isolate [32] and the prevalence of several hvKp biomarkers in its virulome, we can speculate that this isolate may have been linked to invasive disease.

On the other hand, while the local UPKp isolates had traits that were typical of uropathogenic strains including features such as *fimH*, *mrkD*, *iutA*, *feoA/B/C*, *foc*, O1/O2 Variant 2 and O3b O serotypes, and the *usp* protein, H2_41 stood out due to the prevalence of the *ybt* high pathogenicity island. This island was putatively present within an ICE genomic island that also consisted of the T4SS *virB* conjugative machinery. The ICE was predicted based on a minimal blast % ID of 90 and showed similarities to *E*. *coli EDA1* and *K*. *pneumoniae HS11286* among others from the ICEberg dataset. The *ybt* island displayed typical features of a pathogenicity island including (i) a gene cluster size ~32kb, (ii) location next to a tRNA encoding gene (tRNA-Asn-GTT), and (iii) the presence of a gene coding for integrase (*intA*) [71]. This pathogenicity island is commonly found in uropathogenic organisms and incorporates many functions apart from siderophore production and enhancement of bacterial growth. In fact, the *ybt* system also avoids inflammatory responses and the outer membrane receptor *fyuA* contributes to efficient biofilm formation in urine [72, 73]. While we did not obtain clinical data at the time of collection of the cultured isolates, we can postulate that based on this unique trait, this isolate can influence virulence and putatively prolong UTI infection in their host.

Additionally, it was expected that secretion systems were noted in the local isolates since pathogenic *K*. *pneumoniae* use these systems to secrete virulence factors/proteins that can invade the host cell and in turn promote the growth and survival of the pathogen in the host. While the T1SS and T2SS in the local isolates appeared to be involved in the secretions of important proteins such as RTX (Repeats-In-Toxin) cytolysin protein, and pullulanase, the T4SS was responsible for the conjugation and mediating horizontal gene transfer which can contribute to genome plasticity and the basic evolution of infectious pathogens through the dissemination of virulence genes. It is also important to mention that the T6SS, which is considered a versatile weapon used to attack bacterial and fungal competitors and manipulate host cells was observed in the local *K*. *pneumoniae* isolates. The T6SS has become a part of the *K*. *pneumoniae* core genome and is crucial in interspecies and intraspecies competition [74]. This system is especially critical to hvKp and UPKp in transporting proteins, invasion of cells, and most importantly outcompeting other pathogenic species. The local isolates had 12 conserved genes *tss* (B-*M*) which encode the proteins that make up the basic secretion apparatus of a functional T6SS system [75], including the Hcp-VgrG-PAAR structure that transports effector proteins. Herein, the effector proteins noted were similar to those from previously experimentally investigated studies [74, 76–78] and have been noted as playing a role in fungal and bacterial competition. The Tle1^KP^ effector protein from the local isolate was not only 100% identical to the *K*. *pneumoniae* HS11286 strain from which this protein was first detected, but also as expected comprised the G-X-S-X-G motif which belongs to the Tle family. The Tle1^KP^ effector protein has been reported to be involved in periplasmic activity as well as cause growth retardation in neighbouring *E*. *coli* competitors [77], and therefore we can assume that the local isolate may have similar capabilities. We also observed the Tse (EFF01826) effector protein which has been experimentally proven to inhibit the growth of yeast and is therefore directly involved in fungal competition [74, 79]. It is also worth noting that the local isolates had immunity proteins and in turn can protect themselves from lysis/ self-death [74, 80].

Virulence and resistance are the two driving mechanisms that can determine the persistence of infections caused by *K*. *pneumoniae* and more recently, *K*. *quasipneumoniae*. While this study explored the analysis of the virulome and its accompanying mobilome and secretome, resistome analysis is also critical to fully determine the local isolates’ potential from the epidemiological perspective. The unique traits associated with the local hvKp and UPKp isolates aided us in linking these traits to potentially important clinical characteristics. These findings represent features that are now unique to the *K*. *pneumoniae* isolates in Trinidad and thus can be targeted for future surveillance, virulence, and pathogenicity studies in this country as well as in the Caribbean region. While WGS may be prohibitively expensive for hospital laboratories, molecular characterization to determine the prevalence of these traits using PCR of marker genes may be useful to medical practitioners for diagnosis and treating infections caused by *K*. *pneumoniae* and *K*. *quasipneumoniae*.

## Conclusion

*K*. *pneumoniae* is a globally recognized pathogen that can cause severe infections and mortality. The heterogeneity of *K*. *pneumoniae* strains and the potential to disseminate resistant and virulent traits that can promote outbreaks make this pathogen critical in the clinical world. In order to prevent mishaps by this pathogen, it is important to understand the characteristic features associated with such isolates locally. Currently, there is a lack of detailed information on the genomes of clinical Trinidadian *K*. *pneumoniae* isolates. This is the first comprehensive study that investigated the virulome, secretome, and associated mobilome of clinical *K*. *pneumoniae* and *K*. *quasipneumoniae* in Trinidad, West Indies. The data from this study showed that there is a blend of isolates in this country that carried several biomarkers such as adhesion, pili formation, stress tolerance, iron scavenging, CPS, and LPS serotypes that are pathogenically important. While some of the Trinidadian isolates were highly similar to Caribbean and international references, others appeared to be more diverse. More importantly, the local isolates have been shown to be similar to high-risk clones that have caused severe outbreaks internationally. Many different MGEs were positioned around or within the large virulome of the local isolates, thereby suggesting the ease at which dissemination can occur via horizontal gene transfer. Although the data presented in this study suggest that strict infection control measures should be implemented in the health care system, it can also guide medical practitioners during diagnosis and the treatment of infections caused by *K*. *pneumoniae*. The data can also be useful for future genomic studies, especially those focused on investigating the diversity and virulence of isolates in this country.

## Supporting information

S1 Table Origin of local isolates, reference strains, and SNP matrix. (XLSX)

S2 Table Plasmids and phage predicted. (XLSX)

S3 Table Genes associated with siderophores, serotypes, and secretion systems. (XLSX)

S1 Fig COG (A) and KEGG (B) functional annotation of local *K*. *pneumoniae* and *K*. *quasipneumoniae* pangenome elements. (DOCX)

S2 Fig Distribution of insertion sequence elements among local clinical *K*. *pneumoniae* and *K*. *quasipneumoniae* isolates. (DOCX)

S3 Fig Gene organization of virulence factors in local clinical *K*. *pneumoniae* and *K*. *quasipneumoniae* isolates. (DOCX)

## Abbreviations

cKp: Classical *Klebsiella pneumoniae*
cKqp: Classical *Klebsiella quasipneumoniae*
hvKp: Hypervirulent *Klebsiella pneumoniae*
*K. pneumoniae*: *Klebsiella pneumoniae*
*K. quasipneumoniae*: *Klebsiella quasipneumoniae*
UPKp: Uropathogenic *Klebsiella pneumoniae*
