# Supplementary material for: Comparative genomics and virulome analysis reveal unique features associated with clinical strains of Klebsiella pneumoniae and Klebsiella quasipneumoniae from Trinidad, West Indies
Source: PLoS One. 2023 Jul 10;18(7):e0283583. doi: 10.1371/journal.pone.0283583 (PMC10332597; doi:10.1371/journal.pone.0283583)
Supplement: S1 Fig — (DOCX) [file pone.0283583.s004.docx]

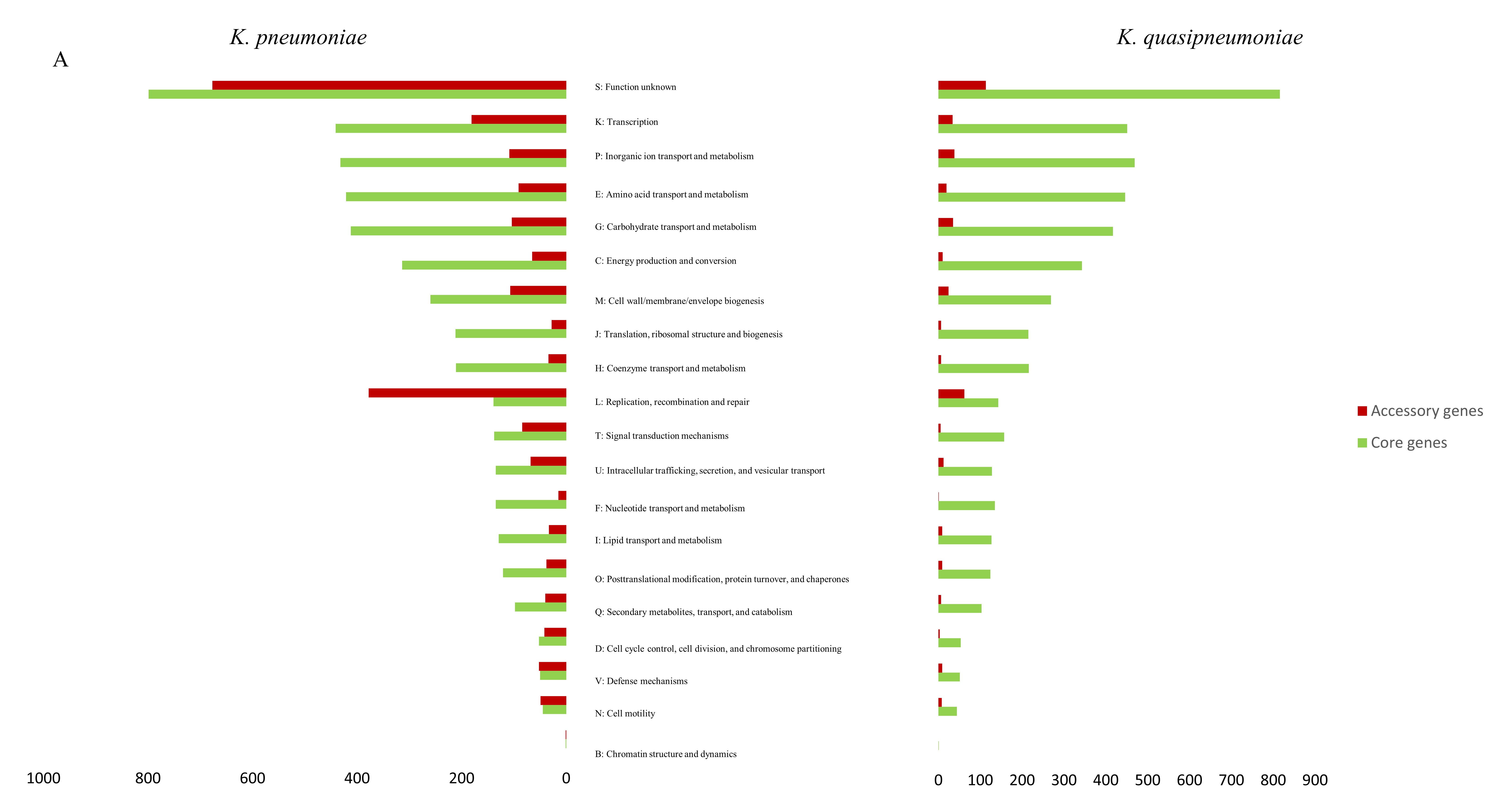


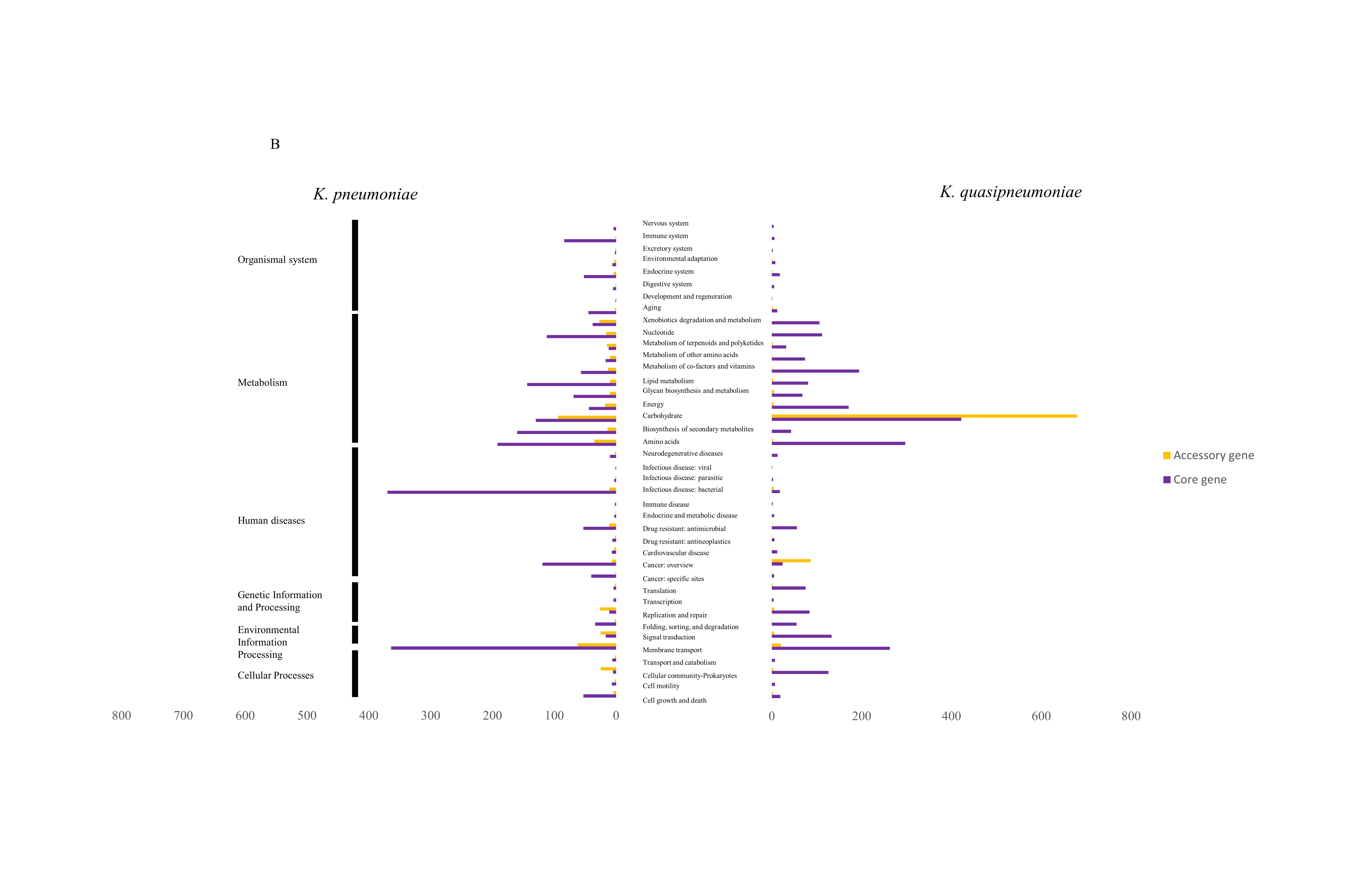


S1 Fig. COG (A) and KEGG (B) functional annotation of local *K. pneumoniae* and *K. quasipneumoniae* pangenome elements.
