# Supplementary material for: Comparative genomics and virulome analysis reveal unique features associated with clinical strains of Klebsiella pneumoniae and Klebsiella quasipneumoniae from Trinidad, West Indies
Source: PLoS One. 2023 Jul 10;18(7):e0283583. doi: 10.1371/journal.pone.0283583 (PMC10332597; doi:10.1371/journal.pone.0283583)
Supplement: S2 Fig — (DOCX) [file pone.0283583.s005.docx]

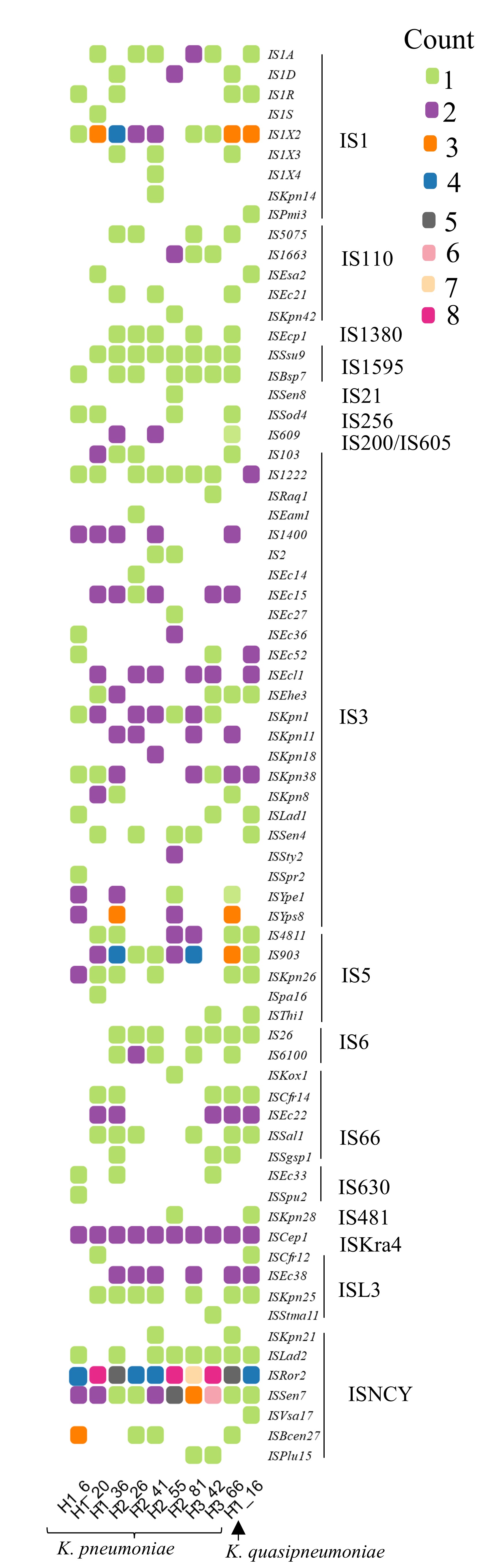


S2 Fig. Distribution of Insertion Sequence elements among local clinical *K. pneumoniae* and *K. quasipneumoniae* isolates. IS elements are grouped according to IS families as indicated in the figure.
