# Supplementary material for: Comparative genomics and virulome analysis reveal unique features associated with clinical strains of Klebsiella pneumoniae and Klebsiella quasipneumoniae from Trinidad, West Indies
Source: PLoS One. 2023 Jul 10;18(7):e0283583. doi: 10.1371/journal.pone.0283583 (PMC10332597; doi:10.1371/journal.pone.0283583)
Supplement: S3 Fig — (DOCX) [file pone.0283583.s006.docx]

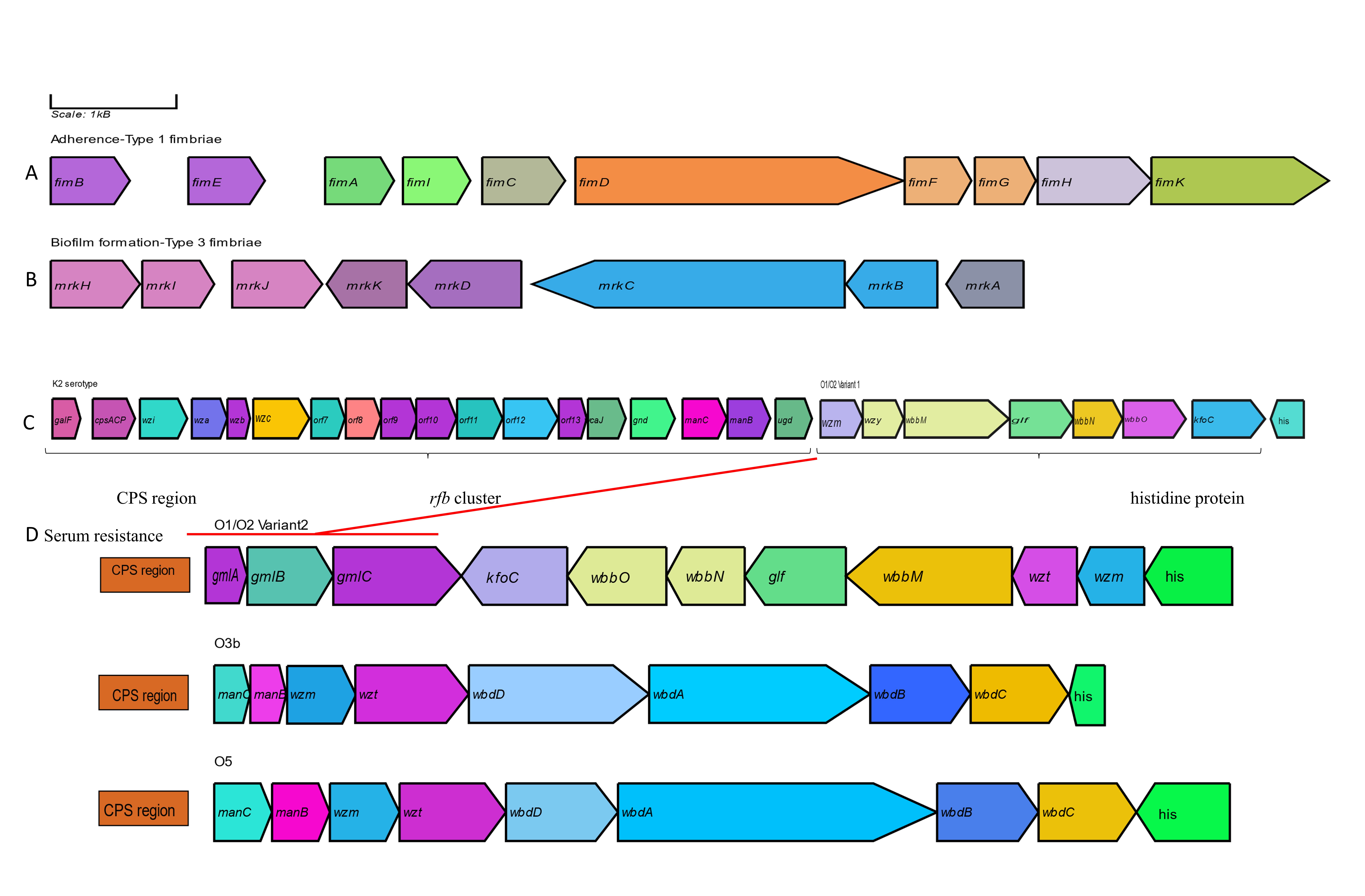


S3 Fig. Gene organization of virulence factors in local clinical *K. pneumoniae* and *K. quasipneumoniae* isolates. A displayed the organization of the Type 1 fimbriae that is responsible for adherence and B displayed the organization of the Type 3 fimbriae that is responsible for biofilm formation. C is the organization of the K2 serotype and the O1/O2 Variant 1 serotype that was found in isolate H2_55. D represents the *rfb* clusters that were observed in the local isolates. The red line indicates the difference in the presence of the *glm* genes in the O1/O2 Variant 2 compared to Variant 1, while the O3b and O5 clusters differed from each other based on the sequence of their methyltransferase (*wbdD*) and mannosyltransferase (*wbdA*) genes*.* The organization of the genes in this figure was generated using Gene Graphics (<https://www.genegraphics.net/>) and further adjustments were done in Inkscape.
